# Serum progranulin levels are associated with frailty in middle-aged individuals

**DOI:** 10.1371/journal.pone.0238877

**Published:** 2020-09-04

**Authors:** Andrew D. Nguyen, Theodore K. Malmstrom, Michael L. Niehoff, Asef Aziz, Douglas K. Miller, John E. Morley

**Affiliations:** 1 Division of Geriatric Medicine, Department of Internal Medicine, Saint Louis University School of Medicine, St. Louis, Missouri, United States of America; 2 Department of Pharmacology and Physiology, Saint Louis University School of Medicine, St. Louis, Missouri, United States of America; 3 Henry and Amelia Nasrallah Center for Neuroscience, Saint Louis University, St. Louis, Missouri, United States of America; 4 Department of Psychiatry and Behavioral Neuroscience, Saint Louis University School of Medicine, St. Louis, Missouri, United States of America; 5 Veterans Affairs Medical Center, St. Louis, Missouri, United States of America; 6 Regenstrief Institute, Inc., Indianapolis, Indiana, United States of America; Raddboudumc, NETHERLANDS

## Abstract

**Background and objective:**

A recent study identified progranulin as a candidate biomarker for frailty, based on gene expression databases. In the present study, we investigated associations between serum progranulin levels and frailty in a population-based sample of late middle-age and older adults.

**Methods:**

We utilized a cohort study that included 358 African Americans (baseline ages 49–65). Frailty was assessed by three established methods: the interview-based FRAIL scale, the Cardiovascular Health Study (CHS) frailty scale that includes performance-based measurements, and the Frailty Index (FI) that is based on cumulative deficits. Serum levels of the following proteins and metabolites were measured: progranulin, cystatin C, fructosamine, soluble cytokine receptors (interleukin-2 and -6, tumor necrosis factor α-1 and -2), and C-reactive protein. Sarcopenia was assessed using the SARC-F index. Vital status was determined by matching through the National Death Index (NDI).

**Results:**

Serum progranulin levels were associated with frailty for all indices (FRAIL, CHS, and FI) but not with sarcopenia. Inflammatory markers indicated by soluble cytokine receptors (sIL-2R, sIL-6R, sTNFR1, sTNFR2) were positively associated serum progranulin. Increased serum progranulin levels at baseline predicted poorer outcomes including future frailty as measured by the FRAIL scale and 15-year all-cause mortality independent of age, gender, and frailty.

**Conclusions:**

Our findings suggest that serum progranulin levels may be a candidate biomarker for physical frailty, independent of sarcopenia. Further studies are needed to validate this association and assess the utility of serum progranulin levels as a potential biomarker for prevalent frailty, for risk for developing incident frailty, and for mortality risk over and above the effect of baseline frailty.

## Introduction

Frailty is a common clinical syndrome in older adults that carries an increased risk for poor health outcomes including falls, incident disability, hospitalization, and mortality [1]. Accurate and robust identification of frailty is important because it allows for appropriate care and interventions. Several methods have been developed and validated to assess frailty. The Cardiovascular Health Study (CHS) frailty scale [2] (which includes two performance-based physical measurements) defines frailty as a distinct clinical syndrome meeting three or more of five criteria: weakness, slowness, low level of physical activity, self-reported exhaustion, and unintentional weight loss. The Frailty Index [3] (FI) assesses frailty as the proportion of cumulative deficits identified in a comprehensive geriatric assessment and is predominantly a comorbidity index. These deficits include diseases (such as cancer, heart failure, cognitive impairment, and others) as well as other self-rated health measures. The interview-based FRAIL scale [4] assesses frailty based on five domains: Fatigue, Resistance, Ambulation, Illnesses, and Loss of weight. The FRAIL scale is a clinic-friendly measure that is both brief and easy to administer as it consists of only five interview questions and does not require measured physical performance. FRAIL consists of key components of the deficit accumulation (FI) and clinical syndrome (CHS) frailty models and is used as a rapid screen. The CHS, FI, and FRAIL methods for assessing frailty have been demonstrated to be effective at predicting disability and mortality [5, 6].

There are currently no validated biomarkers for frailty. Having such frailty biomarkers could complement existing clinical methods for assessing frailty, enable earlier and more robust identification of individuals with frailty, and enable more precise monitoring of frailty status over time. Cardoso *et al*. recently conducted a systematic search to identify candidate biomarkers for frailty based on gene expression databases [7]. They identified progranulin as a key candidate in their proposed core panel of frailty biomarkers. Progranulin is a lysosomal protein of unknown function [8, 9], which is also secreted from cells and is detectable in blood and cerebrospinal fluid [10, 11]. Complete loss of progranulin causes the lysosomal storage disease neuronal ceroid lipofuscinosis [12, 13], whereas haploinsufficiency causes frontotemporal dementia [14, 15]. In the context of these diseases, measurements of progranulin levels in the blood and/or cerebrospinal fluid have provided a reliable method for identifying progranulin mutation carriers [10, 12, 13, 16–19]. Recent studies have found that serum progranulin levels are elevated in a number of diseases including obesity [20, 21], type 2 diabetes [20, 21], kidney disease [22, 23], cancer [24, 25], systemic lupus erythematosus [26, 27], and rheumatoid arthritis [28, 29].

In the current study, we sought to further investigate progranulin as a candidate biomarker for frailty in a cohort study of late middle-aged and older individuals. We investigated the association of serum progranulin with established measures of frailty (CHS, FI, and FRAIL), pro-inflammatory cytokines, and mortality.

## Materials and methods

### Study participants

African American Health (AAH) has been described in previous reports [30, 31]. In summary, the AAH study is a well-characterized population-based cohort study that includes 998 self-identified African American individuals ages 49 to 65 years at baseline in 2000–2001 in the St. Louis, Missouri metropolitan area. Recruitment proportion (participants/enumerated eligible persons) was 76%, and participants completed in-home interviews and assessments at baseline, year 3 follow-up, and year 9 follow-up. Laboratory analyses were done using blood drawn for a subset of participants shortly after the in-home assessments or at the time of clinical examinations at baseline and 3-year follow-up. The final analytic sample for this study includes 358 participants with baseline frailty assessments and serum progranulin level measurements. All study procedures were approved by the Institutional Review Board at Saint Louis University and have therefore been performed in accordance with the ethical standards laid down in the 1964 Declaration of Helsinki and its later amendments.

### Measures

Frailty was assessed as previously described [5] using the International Academy of Nutrition and Aging FRAIL scale [4], the CHS frailty scale [2], and the FI [3]. FRAIL included five items (fatigue, resistance, ambulation, illnesses, loss of weight) with scale scores of 0–5 (one point / item) and represent non-frail (0), pre-frail (1–2) and frail (3–5) health status. The CHS scale included five items (unintentional weight loss, exhaustion, low activity, weakness, and slowness) with scale scores of 0–5 (one point / item) and represent non-frail (0), pre-frail (1–2) and frail (3–5) health status. Our version of the FI included 25 items [5] (0–25; one point / item) with scale scores of 0–1 (total items/25) and represent non-frail (<0.20), pre-frail (0.20–0.25), and frail (>0.25) health status. Sarcopenia was assessed using the SARC-F index (0–10 points; ≥ 4 positive) [32]. Grip strength and gait speed were measured as previously described [32]. Vital status was determined through a National Death Index (NDI) search for calendar years 2000–2014. Deaths are coded as 1.

### Laboratory tests

Serum progranulin levels were determined by Enzyme Linked Immunosorbent Assay (ELISA) (R&D Systems, DPGRN0). Serum samples were diluted 4-fold in diluent buffer and assayed in duplicate. The coefficient of variation (CV) between ELISA plates was 7.2%. Serum Cystatin C levels were determined by ELISA (BioVendor Research and Diagnostic Products). The CV between ELISA plates was 5.5%. Serum fructosamine was measured using a previously described assay [33, 34]. An ELISA kit from ICN-Biomedicals (Costa Mesa, CA) was used to measure soluble Interleukin-6 Receptor (IL-6R); the intra-assay CV was 5.0% and interassay CV was 5.9%. ELISA kits (BioSource, Camarillo, CA) were used to measure soluble Tumor Necrosis Factor Receptor-1 (sTNFR1) (intra-assay CV 4.1%, interassay CV 7.3%) and soluble Tumor Necrosis Factor Receptor-2 (sTNFR2) (intra-assay CV 5.1%, interassay CV 8.6%). C-Reactive Protein (CRP) was measured with a commercially available High-Sensitivity Enzyme Immunoassay (hsCRP ELISA) kit from MP Biomedicals (Orangeburg, NY). The intra-assay and interassay CVs were 4.5% and 4.1%, respectively.

### Statistical approach

Data were analyzed using IBM SPSS Statistics version 19.0 (IBM Corp., Somers, NY). Descriptive statistics are reported as means ± standard deviations (SD) or percentages. Analysis of variance adjusted for age was used to compare baseline serum progranulin for frail classifications (non-frail, pre-frail, frail) on all three measures (FRAIL, CHS, FI), and to compare progranulin levels for chronic diseases (yes/no; kidney disease and diabetes). A paired samples t-test was used to compare baseline versus 3-year follow-up serum progranulin levels. Logistic regression was used to investigate the association of serum progranulin with 15-year all-cause mortality adjusted for age, gender, and frailty. Linear regression adjusted for age was used to investigate the association of serum progranulin with inflammatory cytokines and sarcopenia. Means ± standard deviations are reported for analyses of variance, adjusted odds ratios (ORs) and 95% confidence intervals (CIs) for are reported for logistic regression analyses, and unstandardized (B) regression coefficients and standard errors are reported for linear regression analyses.

## Results

**Fig 1:**
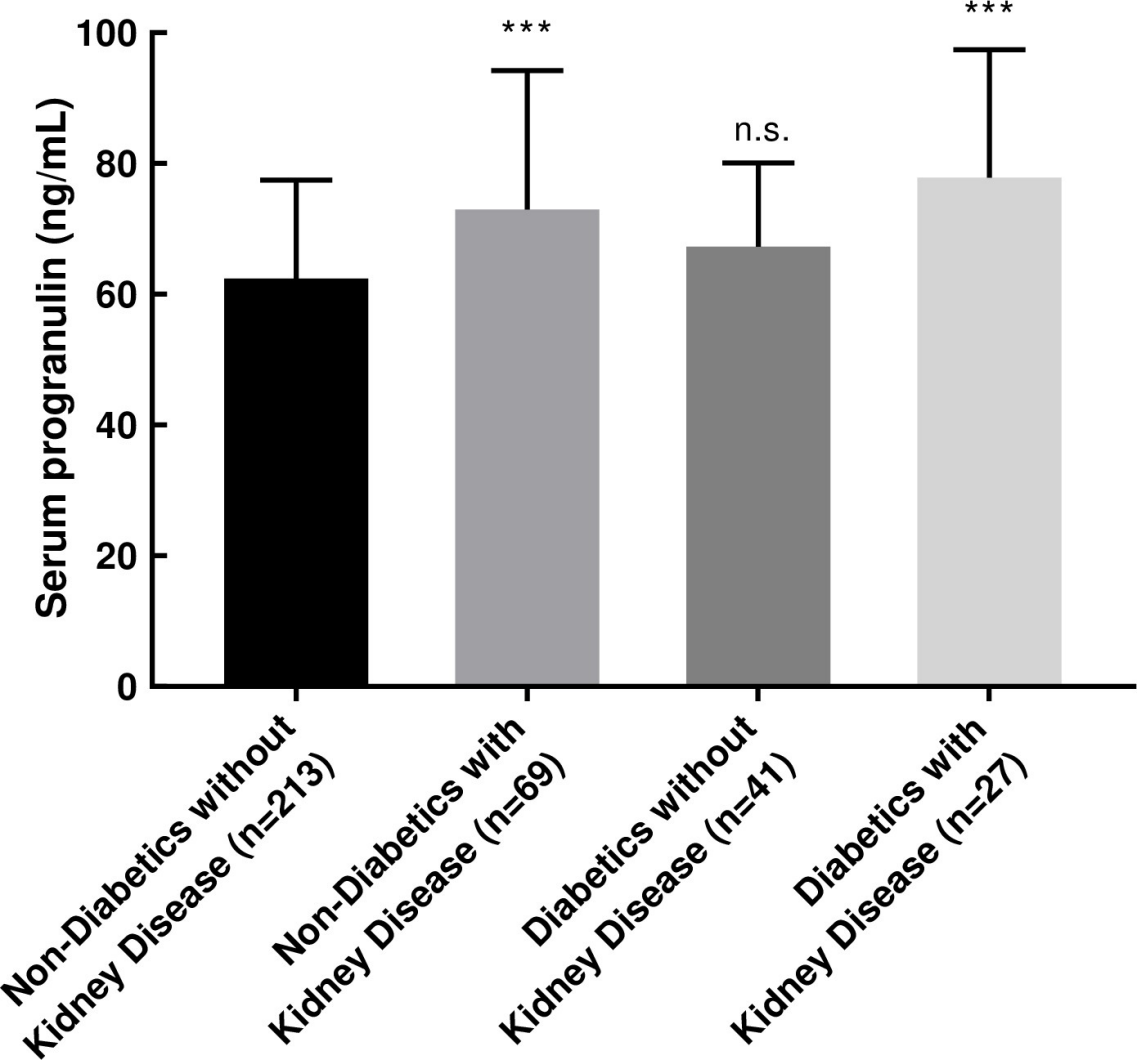
Baseline serum progranulin levels among individuals with diabetes and kidney disease. The criteria for diabetes was fructosamine > 286 μmol/L and for kidney disease was cystatin C > 1.3 mg/L. Statistical methods: ANOVA followed by Tukey post hoc test. The indicated statistical findings represent results from the post hoc tests comparing the indicated group with non-diabetics without kidney disease. ***P < 0.001, n.s., not significant.

Baseline characteristics of the study population are provided in Table 1. Baseline serum progranulin levels (66.33±17.3 ng/mL) are within the range of previous studies [22, 23, 35]. Individuals with kidney disease (27%; cystatin C > 1.3 mg/L) had increased serum progranulin levels (74.32±20.8 ng/mL vs. 63.21±14.8 ng/mL, *P* < 0.001) (S1 Fig), which is consistent with previous findings [22, 23]. Individuals with uncontrolled diabetes (fructosamine > 286 μmol/L) also had increased serum progranulin levels (71.68±16.5 ng/mL vs. 65.05±17.3 ng/mL, *P* = 0.004), but stratification based on cystatin C levels revealed this difference is due to kidney disease co-morbidity (Fig 1). At 3-year follow-up, mean serum progranulin levels increased 7.9% (69.55±16.8 ng/mL vs. 64.47±15.7 ng/mL at baseline, n = 208, *P* < 0.001), supporting a previous report that plasma progranulin levels increase with age [11].

**Table 1:** Baseline characteristics for selected variables.

Characteristic	N	Overall
Age, mean ± SD	358	56.66 ± 4.4
Women	358	66.76%
Diabetes (fructosamine > 286 μmol/L)	355	19.44%
Kidney disease (cystatin C > 1.3 mg/L)	350	27.43%
FRAIL scale (0–5), mean ± SD	347	0.92 ± 1.1
Cardiovascular Health Study (CHS) frailty scale (0–5), mean ± SD	323	0.97 ± 1.1
Frailty Index, mean proportion ± SD	354	0.20 ± 0.2
Serum progranulin (ng/mL), mean ± SD	358	66.32 ± 17.3

At baseline, serum progranulin levels are positively associated with frailty as determined by the FRAIL (*P* = 0.026), CHS (*P* = 0.001), and FI (*P* = 0.005) scales (Table 2). Moreover, serum progranulin levels were higher in the pre-frail group when determined by the FRAIL scale (*P* = 0.006), but not when determined by the CHS (*P* = 0.934) or FI (*P* = 0.182) scales. As serum progranulin levels are elevated in individuals with kidney disease in this (Fig 1 and S1 Fig) and other studies [22, 23], we repeated our analyses excluding individuals with kidney disease; serum progranulin levels remained positively associated with frailty as determined by the FRAIL scale (*P* = 0.031) (S1 Table). Excluding subjects frail at baseline, increased baseline serum progranulin levels are also positively associated with higher FRAIL scores at 9-year follow-up (*P* = 0.006); associations were not observed with the other frailty scales (CHS: *P* = 0.241, FI: *P* = 0.246), likely due to the smaller sample sizes for these groups. Baseline serum progranulin levels adjusted for age, gender, and frailty scores also were associated with all-cause mortality over the 15-year follow-up (Table 3).

**Table 2:** Baseline serum progranulin and frailty classification.

Measure	Progranulin (ng/mL)	N	*P*
International Academy of Nutrition and Aging (FRAIL) frailty scale			F = 4.92, *P* = 0.008^a^
Non-frail (0)	63.45 ± 16.68	167	
Pre-frail (1–2)	68.80 ± 17.48	141	0.006^b^
Frail (3–5)	70.57 ± 18.20	39	0.026^b^
Cardiovascular Health Study (CHS) frailty scale			F = 5.78, *P* = 0.003^a^
Non-frail (0)	65.22 ± 17.44	142	
Pre-frail (1–2)	65.08 ± 15.02	152	0.934^b^
Frail (3–5)	76.74 ± 24.37	29	0.001^b^
Frailty Index (FI)			F = 3.99, *P* = 0.019^a^
Non-frail (<0.20)	63.55 ± 15.92	150	
Pre-frail (0.20–0.25)	66.69 ± 17.60	85	0.182^b^
Frail (>0.25)	69.73 ± 18.35	119	0.005^b^

^a^ Univariate ANOVA adjusted for age

^b^ Contrast with reference category non-frail group

**Table 3:** Baseline serum progranulin and 15-year all-cause mortality.

Independent Variable	Odds Ratio (95% CI)	*P*
		N = 347
Age	1.13 (1.07–1.20)	<0.001
Female	0.61 (0.35–1.04)	0.068
FRAIL scale total (0–5)	1.32 (1.06–1.66)	0.015
Serum progranulin	1.02 (1.01–1.03)	0.008
		N = 323
Age	1.15 (1.08–1.23)	<0.001
Female	0.61 (0.35–1.06)	0.080
CHS frailty scale total (0–5)	1.12 (0.90–1.39)	0.324
Serum progranulin	1.02 (1.01–1.04)	0.006
		N = 354
Age	1.13 (1.07–1.20)	<0.001
Female	0.57 (0.34–0.97)	0.039
Frailty Index (0–1)	12.22 (3.02–49.50)	<0.001
Serum progranulin	1.02 (1.01–1.03)	0.010

Serum progranulin levels adjusted for age were associated with pro-inflammatory markers including soluble forms of TNFR1, TNFR2, Interleukin-2 Receptor (IL-2R), and IL-6R (Table 4), but not with CRP; these findings are largely consistent with correlations between levels of serum progranulin and inflammatory markers reported in previous studies [20, 21, 23]. Progranulin levels did not correlate with sarcopenia as determined by the SARC-F index [32] (unstandardized beta = 0.65, SE = 0.42, *P* = 0.13). Additionally, progranulin levels did not significantly correlate with grip strength (*P* = 0.100) or gait speed (*P* = 0.087). Together, our results suggest that the increased serum progranulin levels observed in frail individuals is independent of sarcopenia and may implicate inflammation as part of the causal chain.

**Table 4:** Baseline serum progranulin and cytokine levels.

Measure	N	Unstandardized Beta (SE)	*P*^a^
Progranulin			
CRP	353	0.210 (0.128)	0.101
CRP (log_10_)	353	0.636 (1.182)	0.726
sIL-2R	353	0.020 (0.002)	<0.001
sIL-2R (log_10_)	353	25.365 (3.643)	<0.001
sIL-6R	352	0.175 (0.038)	<0.001
sIL-6R (log_10_)	352	18.797 (5.054)	<0.001
TNFR1	351	1.621 (0.197)	<0.001
TNFR1 (log_10_)	351	32.622 (4.152)	<0.001
TNFR2	351	0.737 (0.078)	<0.001
TNFR2 (log_10_)	351	37.803 (3.791)	<0.001

^a^ Ordinary Least Squares Regression adjusted for age.

## Discussion

We found a positive association between serum progranulin levels and both prevalent and incident frailty in late middle-aged and older individuals. Serum progranulin levels were associated with frailty as determined by three independent measures (FRAIL, CHS frailty scale, and FI). The positive associations with the FRAIL and CHS measures suggest that serum progranulin levels are associated with physical frailty. SARC-F, grip strength, and gait speed were not significantly associated with progranulin levels. Longitudinally, increased serum progranulin levels at baseline predicted poorer outcomes including future frailty as determined by the FRAIL scale and all-cause mortality.

The reason for this association between serum progranulin levels and frailty is unclear. Circulating progranulin levels are increased in a number of diseases, including kidney disease [22, 23], obesity [20, 21], type 2 diabetes [20, 21], cancer [24, 25], systemic lupus erythematosus [26, 27], and rheumatoid arthritis [28, 29]. In many of these conditions, the increased progranulin levels are correlated with increased inflammatory markers [20–22, 27]. In kidney disease, circulating progranulin levels are elevated due to impaired renal clearance of progranulin [22, 23]. Consistent with this, we observed increased serum progranulin levels in individuals with kidney diseases in our study (Fig 1 and S1 Fig). Therefore, we performed our analysis for frailty both including and excluding individuals with kidney disease. In both cases, we detected associations between serum progranulin levels and frailty as determined by the FRAIL scale (Table 2 and S1 Table). Progranulin levels are regulated by inflammation [36, 37] as well as by lysosomal transcription factors [38, 39]. Since inflammation and lysosome biology are both tightly linked with aging [40, 41], it is possible that these processes contribute both to the development of frailty and to the increased serum progranulin levels. In support of this, we found strong correlations between serum progranulin levels and a small panel of pro-inflammatory markers (soluble forms of TNFR1, TNFR2, IL-2R, and IL-6R), with the exception of CRP, suggesting the increased serum progranulin levels may be in part due to increased inflammation. In this study, we measured levels of soluble cytokine receptors, rather than the cytokines, because changes in the soluble cytokine receptors are more prolonged and therefore reflect chronic inflammation [42].

In a recent study seeking to identify candidate biomarkers for frailty, Cardoso *et al*. identified progranulin as a potential frailty biomarker, based on gene expression data from databases [7]. In support of this, our study provides evidence at the protein level that serum progranulin levels are positively associated with frailty. Specifically, our results suggest that serum progranulin levels may be a candidate biomarker for prevalent physical frailty and a risk factor for incident frailty, independent of sarcopenia. While the precise function of progranulin is still under investigation, other studies have established that circulating progranulin levels are influenced by a number of disease processes, thereby limiting the specificity of circulating progranulin levels as a biomarker for frailty. We envision that progranulin may serve as one component in a panel of biomarkers for frailty, as previously proposed [7].

A study by Woo et al. found that approximately 25% of frail individuals were not sarcopenic [43]. Other studies have similarly found a subset of frail persons do not have sarcopenia [44]. In our study, we did not find associations between progranulin levels and the SARC-F or other correlates of sarcopenia (gait speed, grip strength). This suggests that other causes may play a role in frailty that are not due to sarcopenia and may account for the lack of an association of sarcopenia and progranulin levels.

Genetic studies have established that reduced progranulin levels cause the neurodegenerative diseases frontotemporal dementia [14, 15] and neuronal ceroid lipofuscinosis [12, 13]. Several studies have suggested that low progranulin levels may also be a risk factor for Alzheimer’s disease [10, 45, 46], and mild cognitive impairment [17, 47]. In our study, we did not find associations between serum progranulin levels and the limited cognitive tests performed, including Mini-Mental State Examination (MMSE), Animal Naming Test, and Trail Making Tests A and B (data not shown). One possible reason is that serum progranulin levels may not accurately reflect progranulin levels in the central nervous system, as previously reported [11]. Further investigation is warranted, particularly in longitudinal studies with more comprehensive cognitive testing and/or biomarkers of neurodegeneration.

This study has limitations. Since the participants were predominantly late middle-aged and older African American community-dwelling population living in a restricted geographic area, these results may not generalize to other populations. Another limitation of this study is the analysis of a single biomarker; future studies should incorporate measurements of additional biomarkers and should also take into account the cognitive and psycho-social dimensions of frailty. The major strengths of this study include the use of multiple validated measurements of frailty and the use of sensitive biochemical measurements as criteria for many of the diseases, including kidney disease and diabetes.

In summary/conclusion, we found a positive association between serum progranulin levels and frailty in late middle-aged and older individuals. While our findings suggest that serum progranulin levels may be a useful biomarker for physical frailty independent of sarcopenia, further studies are needed to validate these associations and assess the utility of serum progranulin levels as a potential biomarker for frailty.

## Supporting information

S1 Fig Baseline serum progranulin levels among individuals with kidney disease. The criterion for kidney disease was cystatin C > 1.3 mg/L. ***P < 0.001.
(PDF)

S1 Table Baseline serum progranulin and frailty (excluding persons with cystatin C > 1.3 mg/L). (DOCX)
