# Supplementary figures and images for: Serum progranulin levels are associated with frailty in middle-aged individuals
Source: PLoS One. 2020 Sep 4;15(9):e0238877. doi: 10.1371/journal.pone.0238877 (PMC7473561; doi:10.1371/journal.pone.0238877)

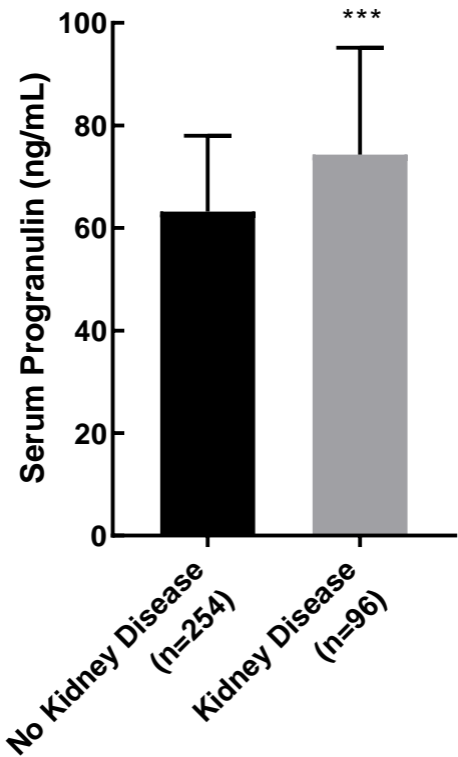

**S1 Figure**

Supplement: S1 Fig — The criterion for kidney disease was cystatin C > 1.3 mg/L. ***P < 0.001. (PDF) [file pone.0238877.s001.pdf]
