# Supplementary material for: Serum progranulin levels are associated with frailty in middle-aged individuals
Source: PLoS One. 2020 Sep 4;15(9):e0238877. doi: 10.1371/journal.pone.0238877 (PMC7473561; doi:10.1371/journal.pone.0238877)
Supplement: S1 Table — (DOCX) [file pone.0238877.s002.docx]

| **S1 Table.** Baseline serum progranulin and frailty (excluding persons with cystatin C > 1.3 mg/L) | | | | | | | | | | | |
| --- | --- | --- | --- | --- | --- | --- | --- | --- | --- | --- | --- |
| Measure | | | Progranulin (ng/mL) | | | | N | | *P* | |  |
|  | | | | | | | | | | | |
| International Academy of Nutrition and Aging (FRAIL) frailty scale F=4.23, *P*=0.016^a^ | | | | | | | | | | | |
| Non-frail (0) | | | 60.73 ± 14.97 | | | | 125 | |  | |  |
| Pre-frail (1-2) | | | 65.50 ± 13.08 | | | | 99 | | 0.015^b^ | |  |
| Frail (3-5) | | | 68.14 ± 19.20 | | | | 23 | | 0.031^b^ | |  |
| Cardiovascular Health Study (CHS) frailty scale F=1.30, *P*=0.275^a^ | | | | | | | | | | | |
| Non-frail (0) | | | 62.53 ± 16.07 | | | | 107 | |  | |  |
| Pre-frail (1-2) | | | 62.77 ± 12.86 | | | | 107 | | 0.917^b^ | |  |
| Frail (3-5) | | | 69.37 ± 18.17 | | | | 15 | | 0.115^b^ | |  |
| Frailty Index (FI) F=1.18, *P*=0.309^a^ | | | | | | | | | | | |
| Non-frail (<0.20) | | | 61.80 ± 14.94 | | | | 114 | |  | |  |
| Pre-frail (0.20-0.25) | | | 63.42 ± 15.43 | | | | 61 | | 0.508^b^ | |  |
| Frail (>0.25) | | | 63.26 ± 14.83 | | | | 76 | | 0.127^b^ | |  |
|  | |  | | |  | | |  | | | |
|  |  | | |  | |  | | | |  |  |

^a^ Univariate ANOVA adjusted for age

^b^ Contrast with reference category non-frail group
